# Evaluation of IVOCT imaging of coronary artery metallic stents with neointimal coverage

**DOI:** 10.1007/s10554-014-0569-7

**Published:** 2014-11-14

**Authors:** Sahar Elahi, Derek Ho, Marc D. Feldman, Jouke Dijkstra, Thomas E. Milner

**Affiliations:** 1 Department of Biomedical Engineering, University of Texas at Austin, 107 W Dean Keeton Street Stop C0800, Austin, TX 78712 USA; 2 Division of Cardiology, School of Medicine, University of Texas Health Science Center at San Antonio, San Antonio, TX USA; 3 Department of Radiology, Leiden University Medical Center, Leiden, The Netherland

**Keywords:** IVOCT, Stent, Neointima, Merry-go-round

## Abstract

Accuracy of IVOCT for measurement of neointimal thickness and effect of neointima in the appearance of metallic struts in IVOCT images was investigated. Phantom vessels were constructed and coronary stents were deployed and covered with thick (250–400 μm) and thin (30–70 μm) phantom neointima. High resolution Micro-CT images of the stent struts were recorded as a gold standard. IVOCT images of the phantom vessels were acquired with various luminal blood scattering strengths and measured neointimal thicknesses from IVOCT and Micro-CT images were compared. In transparent lumen, comparison of IVOCT and Micro-CT neointima thickness measurements found no significant difference (*p* > 0.05) in the thick neointima phantom but a significant difference (*p* < 0.05) in the thin neointima phantom. For both thick and thin neointima, IVOCT neointimal thickness measurements varied from Micro-CT values by as much as ±35 %. Increased luminal scattering due to presence of blood at concentrations <5 % did not interfere with measurement of thin neointimas and was validated by ANOVA analysis (*p* = 0.95). IV-OCT measurement of strut feature size with an overlying thin neointima match true values determined with Micro-CT (*p* = 0.82). Presence of a thick neointima resulted in lateral elongation or merry-go-rounding of stent strut features in IVOCT images. Phantom IVOCT images suggest that thick neointimal layers can result in more than 40 % lateral elongation of stent strut features. Simulated IVOCT images of metallic stent struts with varying neointimal thickness suggest that neointimal light scattering can introduce the merry-go-round effect.

## Introduction

Formation of neointima after stent deployment is an important indicator of the vascular healing process. While the presence of neointima is desired in the healing response, if the neointima thickens excessively re-stenosis results—a frequent complication with bare metal stents. To prevent re-stenosis, drug-eluting stents limit neointimal formation by releasing immunosuppressant pharmaceuticals which inhibit smooth muscle cell proliferation. Cypher (Cordis Corp., Miami Lakes, Florida) and Taxus (Boston Scientific Corp., Natick, Massachusetts) stents resulted in delayed neointimal formation when compared with bare metal stents of similar implant duration [1]. These findings have been extended to second-generation drug eluting stents [2].

Human autopsy studies suggest that the lack of neointimal strut coverage due to delayed vascular healing is associated with acute stent thrombosis [3]. Therefore, accurate in vivo assessment of neointimal formation after stenting during long term follow-up may aid in the identification of patients at risk for late stent thrombosis.

Intravascular Optical Coherence Tomography (IVOCT) with high axial resolution (15 μm) and tissue penetration depth of 1.5–2.0 mm offers the best imaging technology to assess the neointimal thickness compared to e.g. Intravascular Ultrasound [4–6]. However, clinical interpretation of a neointimal thickness that is or near the axial resolution of the IVOCT system is not well developed. Moreover, impact of luminal scattering due to residual blood on neointimal thickness measurement has not been investigated. In cases where struts may have partial tissue coverage, some experts consider the strut as covered, whereas others suggest that these struts be classified as having incomplete coverage [7, 8]. A study on the accuracy of IVOCT in analyzing the neointimal response to several drug-eluting stents, showed significant variation in the estimation of strut coverage between IVOCT and histology when the neointimal thickness was between 20 to 80 μm [9] which is the range of thicknesses corresponding to thin neointimas. Due to the known artifact of tissue shrinkage in histology, phantoms will provide a more accurate standard for neointimal thickness measurement than human autopsy specimens. One objective of the present study was to evaluate the accuracy IVOCT thickness measurements of thick and thin neointimas in a phantom vessel.

Luminal scattering caused by residual blood that was incompletely flushed and/or formation of micro-bubbles when contrast is injected into the lumen can impact clinical IVOCT images. As increased luminal scattering reduces intensity of detected light and results in loss of fine image details, the effect could impact measurement of thin neointimal layers. For thicker neointimal layers, increased luminal scattering may influence apparent strut feature size. A second objective of this study is to evaluate the accuracy of neointimal thickness measurements by IVOCT in presence of varying luminal scattering strengths due to residual blood contamination. The impact of thick neointima on the longitudinal size of strut features is also characterized as a new etiology for the merry-go-round artifact [10].

## Methods

### Phantom vessel fabrication

**Fig. 1:**
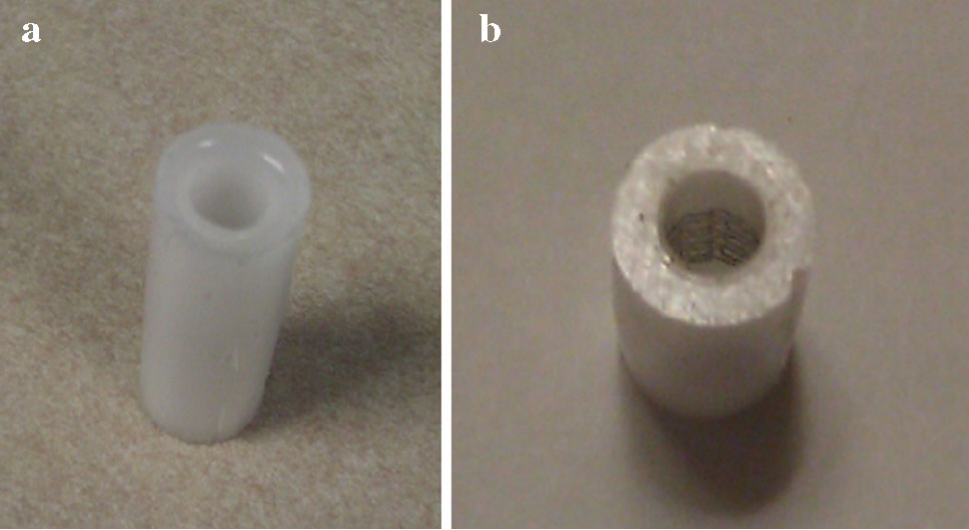
**a** PDMS phantom vessel after removal from mold, **b** deployed stent in phantom vessel

A mold consisting of a 3 mm inner-diameter brass cylinder positioned inside an outer cylindrical aluminum housing (6 mm diameter × 25 mm length) was constructed to make phantom vessels. To register IVOCT and Micro-CT images (see below) a 125 μm diameter glass optical fiber oriented parallel to the long axis was embedded in the phantom vessel and used as an azimuthal reference marker. Two phantom vessels were made by pouring polydimethylsiloxane (PDMS) mixed with titanium dioxide into the mold. After curing, the phantom vessel was removed from the mold and 3 × 8 mm TAXUS^®^ Liberte^®^ stents were deployed at a balloon pressure of 16 atm for 30 s (Fig. 1).

### Addition of phantom neointimal coverage

Neointima was added to phantom vessels, giving a thin and a thick layer. A two-piece aluminum cylinder, coated with Teflon, was inserted into each phantom vessel and a mix of PDMS and titanium dioxide was injected into the space between aluminum cylinder and vessel wall to form a neointima. For thin neointima where thickness was <50 μm, a scattering coefficient of 12.7 cm^−1^ was taken for early after stenting—<1 day—which is similar to the cellular epidermal layer in skin [11]. For thick neointima where thickness was <450 μm, a scattering coefficient of 8.1 cm^−1^ was taken for long-term stenting—more than 2 weeks—similar to fibrous dermal layer in skin [11]. After curing, the two halves of the aluminum cylinder were removed, leaving a phantom neointima covering the deployed metallic stent.

### Micro-CT imaging

Micro-CT images of TAXUS^®^ Liberte^®^ stents deployed in the phantom vessels with neointima were acquired and utilized as “gold standard” to determine stent struts’ size and neointimal thickness. For each slice, 1,000 views were recorded and field size of image reconstruction was 6 × 6 mm^2^. Each image slice was rendered at 1,024 × 1,024 pixel resolution, resulting in an in-plane resolution of 5.86 µm per pixel. When imaging thin neointima, higher power was used to achieve a resolution of 3.5 µm (both in-plane and interslice).

### IVOCT imaging

Images were acquired using a frequency domain IVOCT system (CorVue, Volcano Corporation) while the phantom vessel was flushed with saline. The CorVue system operated at a 1,310 nm center wavelength with a 12 μm axial resolution at an A-scan rate of 20 kHz. Catheter was pulled back at a slow speed of 1.5 mm/s over a 15 mm length of vessel and frame rate of 30/s To study the effect of luminal scattering strength on measurement of thin neointimas, goat blood was mixed with saline at concentrations of 0.5, 1.0, 2.0 and 5.0 %. The mix was injected by a syringe while the IVOCT catheter was pulled back through the phantom.

### IVOCT/Micro-CT image registration

Image registration between IVOCT and Micro-CT data sets was performed in MATLAB (Mathworks, R2013a). Since IVOCT frames were recorded at 50 μm intervals, a single IVOCT frame corresponded to 9 Micro-CT sequential images of thick neointima and 15 sequential images of thin neointima. Longitudinal registration was completed by comparing the subset of Micro-CT images corresponding to a given IVOCT image. The 125 μm diameter glass optical fiber oriented parallel to the long axis was visible in both IVOCT and Micro-CT images and used as a marker for azimuthal registration. Following image registration, neointima thickness and strut size feature measurements derived from IVOCT and Micro-CT images were performed manually in MATLAB (Mathworks, R2013a). For both thin and thick neointima, thickness taken from IVOCT images was measured from the middle of the strut feature to the luminal wall. Neointimal thickness determined from Micro-CT images was measured from the proximal edge of the strut feature to the luminal wall.

### Statistical analysis

Statistical analysis was performed using paired *t* test to compare the neointimal thickness measurements of IVOCT and Micro-CT images as well as the strut feature size measurements of IVOCT and Micro-CT images for both thick and thin neointima phantoms. For both paired t-tests, the null hypothesis is that the IVOCT and Micro-CT measurement means are equivalent. Analysis of variance (ANOVA) was performed to compare strut feature size for four luminal blood scattering strengths (0.5, 1.0, 2.0 and 5.0 %) in the thin neointima phantom. Twenty independent measurements of neointimal thickness in IVOCT images for each luminal blood scattering strength were measured. For ANOVA tests, the null hypothesis is that mean neointimal thickness for each luminal blood scattering strength are equivalent.

### Simulation of neointima coverage

Elahi et al. [12] has described a computational model of an IVOCT catheter and a metallic stent strut using optical design software (ZEMAX, Radiant, Seattle, WA). In the present study, the same IVOCT catheter model was employed and the strut was covered with a 50 μm scattering layer simulating thin neointimal coverage; where refractive index (*n*) of 1.42 and scattering coefficient (μ_*s*_) of 12.7 cm^−1^ were assumed for early neointima (epidermis). For the thick neointima (400 μm) *n* = 1.37 and μ_*s*_ = 8.1 cm^−1^ corresponding to late neointima (dermis) [11, 13].

## Results

### Accuracy of neointimal thickness measurement

**Fig. 2:**
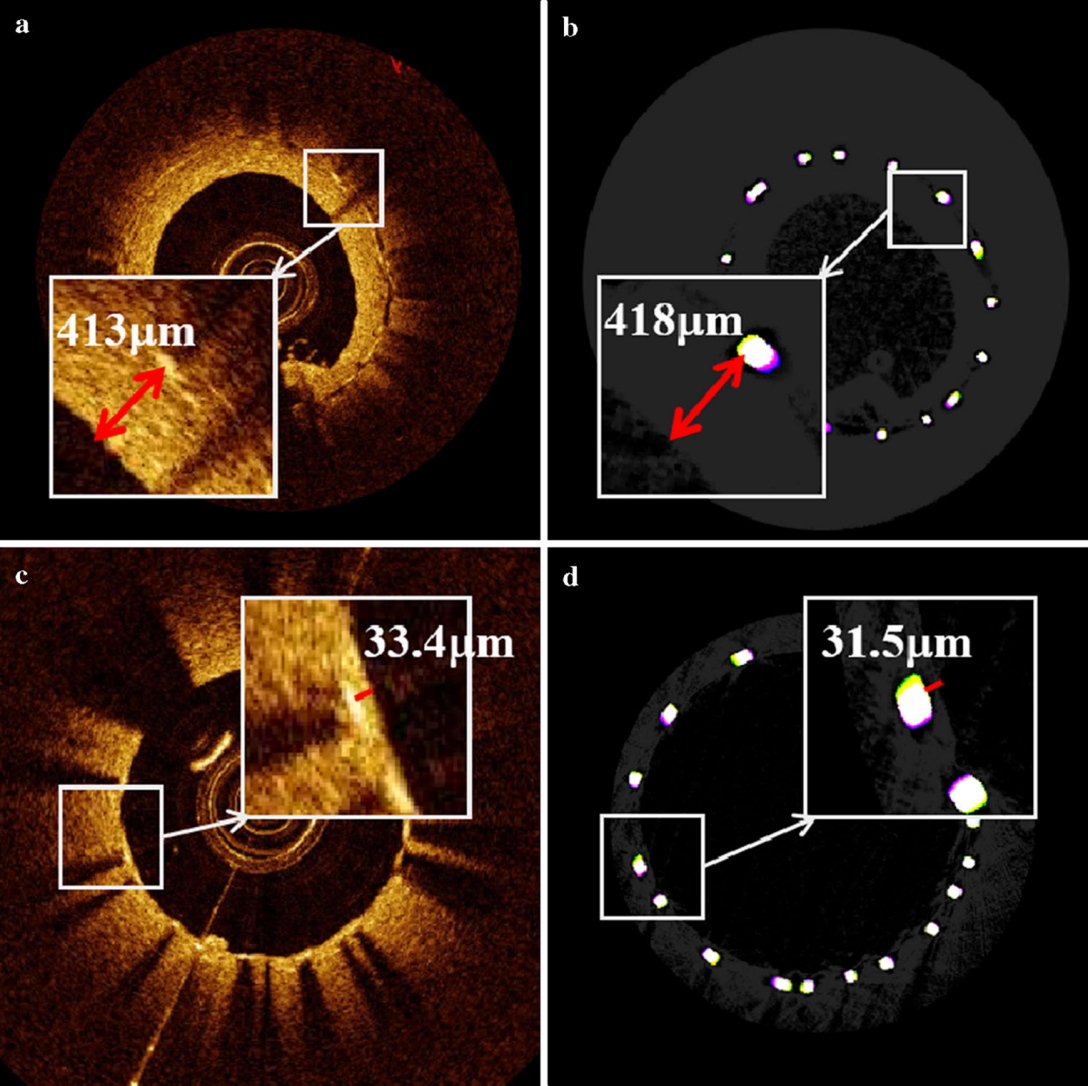
Neointimal thickness measurement; thick neointima by **a** IVOCT and **b** Micro-CT and thin neointima by **c)** IVOCT and **d** Micro-CT

Measurements of neointimal thickness from IVOCT images were compared to values obtained from Micro-CT images which show the true neointimal thickness. Examples of thickness measurement for thick and thin neointimas are given in Fig. 2 where we were able to measure the thickness of neointima in IVOCT images as thin as 30 μm accurately.

Thickness measurement was completed for 141 IVOCT frames of thick neointima and 25 IVOCT frames of thin neointima (in some cases phantom neointima did not cover the entire stent) and the corresponding Micro-CT images, given in Table 1. To obtain IVOCT neointimal thickness, measured IVOCT optical pathlengths were divided by refractive index of PDMS (*n* = 1.405) [14].

**Table 1:** Neointima thickness measurements: m is mean, ρ is standard deviation and ε is fractional error in IVOCT measurements normalized against Micro-CT (141 measurements for thick neointima and 25 measurements for thin neointima)

Thin neointima	Thick neointima
*IVOCT*	
m = 49.30 μm	m = 319.63 μm
ρ = 12.27 μm	ρ = 48.10 μm
ε = 29 %	ε = 5 %
*Micro*-*CT*	
m = 38.22 μm	m = 305.38 μm
ρ = 9.44 μm	ρ = 61.13 μm

**Fig. 3:**
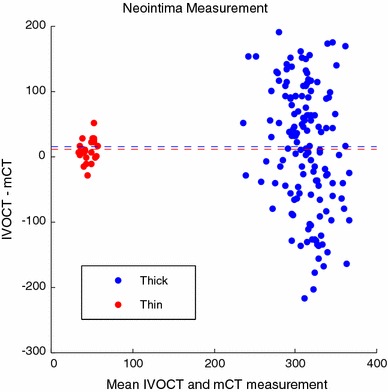
Bland-Altman plot of IVOCT and Micro-CT (mCT) thickness measurements for thin (*red*) and thick (*blue*) neointima. *Horizontal lines* represent mean differences

A Bland–Altman plot was constructed to display differences between thickness measurement of thin and thick neointima using IVOCT and Micro-CT (Fig. 3). Mean difference between neointimal thickness measured with IVOCT and Micro-CT for both thick (15 μm) and thin (11 μm) neointima exhibit a non-negative offset.

Paired t-test rejected the null hypothesis for thin neointima (*p* = 0.003) and failed to reject the null hypothesis for thick neointima (*p* = 0.08).

### Effect of blood luminal scattering on measurement of thin neointimal layers

**Fig. 4:**
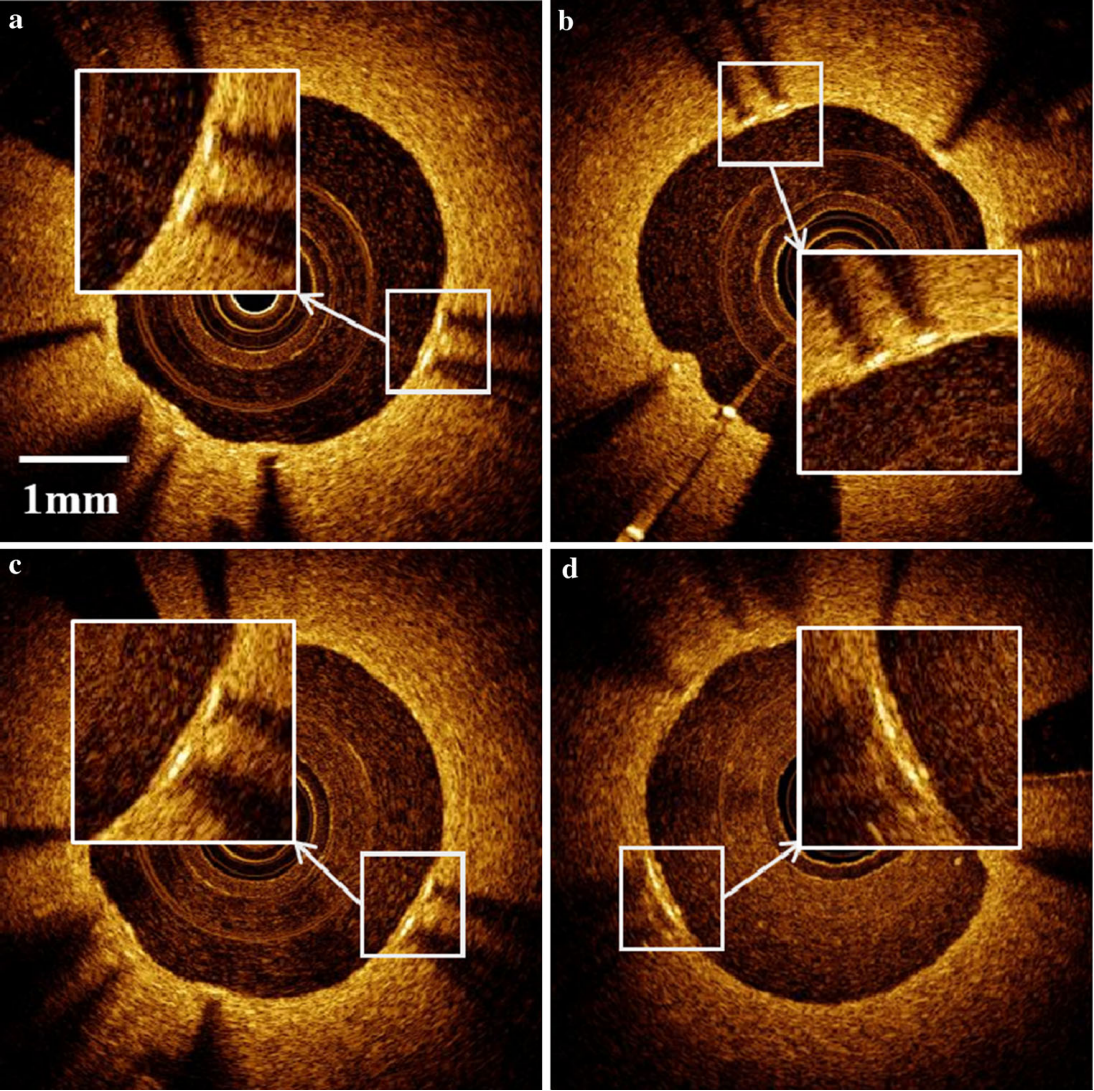
Increasing luminal scattering strength did not impact thickness measurement of thin neointimas when flushed with blood concentrations of: **a** 0.5 %, **b** 1 %, **c** 2 % and **d** 5 %

IVOCT images of the phantom vessel with a stent deployed with thin neointimal coverage were acquired where luminal scattering strength was varied by different blood-saline mixes (0.5, 1.0, 2.0 and 5.0 %). As suggested in Fig. 4, increased blood concentrations increased luminal scattering, however, the presence of up to 5 % blood does not appear to affect thickness measurement of thin neointimas. ANOVA analysis failed to reject the null hypothesis (*p* = 0.96) which suggests that mean neointimal thicknesses measured with IVOCT are independent of luminal scattering strength over the investigated blood concentrations.

### Merry-go-round effect due to neointima

**Fig. 5:**
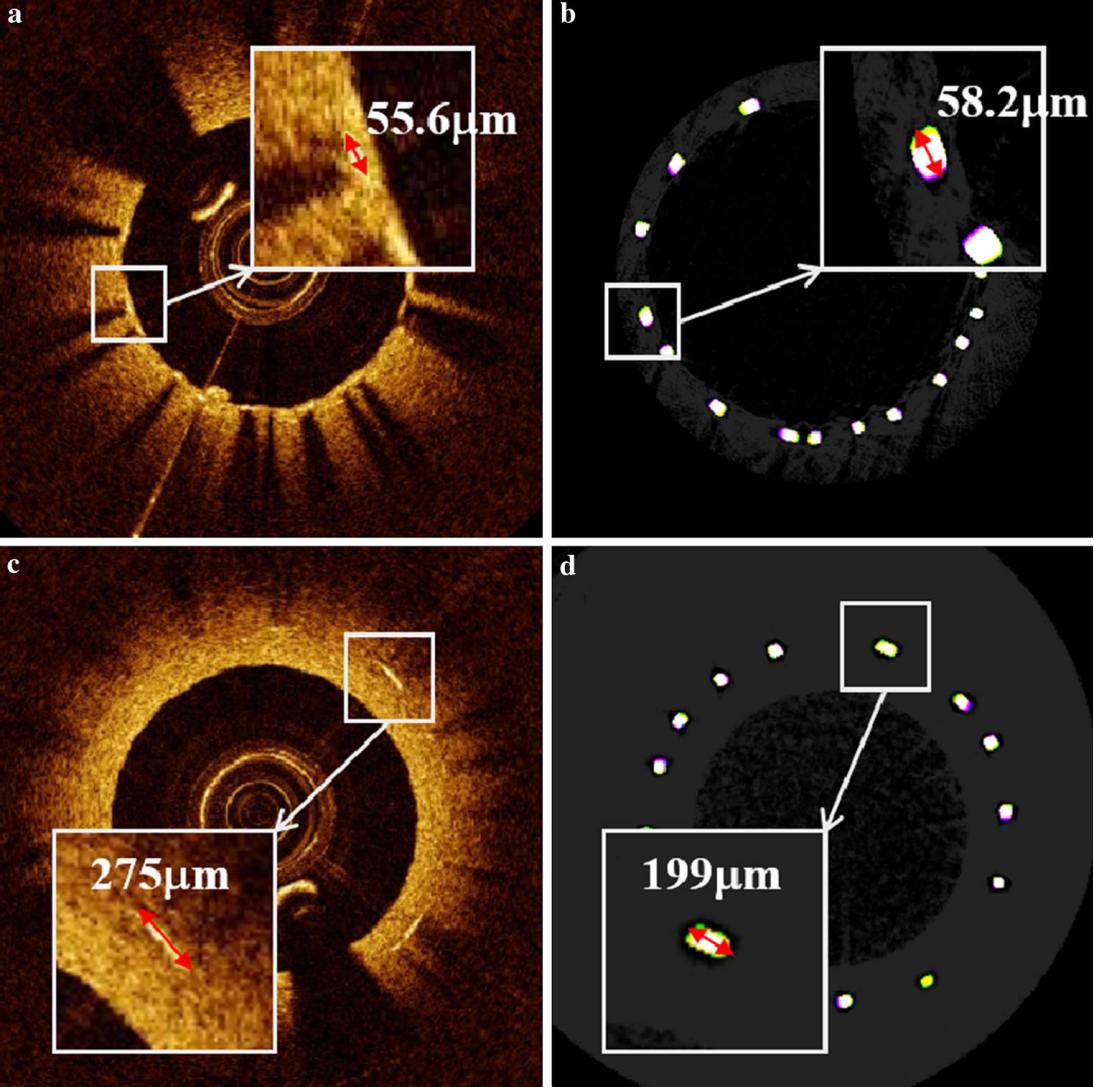
Effect of neointima on merry-go-round artifact; **a** IVOCT and **b** Micro-CT images of thin (33 μm) neointima, **c** IVOCT and **d** Micro-CT images of thick neointima (390 μm) where merry-go-round artifact is observed

Scattering by thick neointimas resulted in elongation (“merry-go-round”) of strut features in IVOCT images. Figure 5 illustrates examples of strut feature size (length of red double-sided arrows) in presence of thick and thin neointimas.

Measurement of strut size from IVOCT and Micro-CT images was compared (Table 2). Relatively high standard deviation in measurement results from variation in strut size. No correlation was observed between the strut size and neointimal thickness in the range of 200–400 μm, consistent with marked “merry-go-round” artifact with thick neointima.

**Table 2:** Strut size measurements: m is mean, ρ is standard deviation and ε is fractional error in IVOCT measurements normalized against Micro-CT

Thin neointima	Thick neointima
*IVOCT*	
m = 215.79 μm	m = 205.94 μm
ρ = 89.60 μm	ρ = 74.81 μm
ε = −1.5 %	ε = 42 %
*Micro*-*CT*	
m = 219.22 μm	m = 145.30 μm
ρ = 112.32 μm	ρ = 68.53 μm

**Fig. 6:**
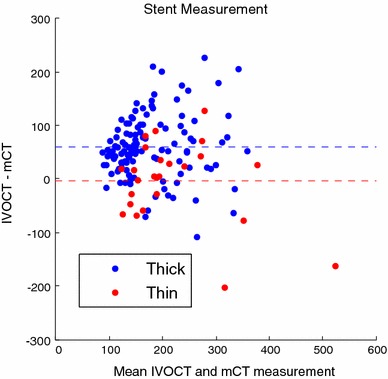
Bland-Altman plot of IVOCT and Micro-CT (mCT) thickness measurements for thin (*red*) and thick (*blue*) neointima. *Horizontal lines* represent mean differences

A Bland–Altman plot was constructed to investigate IVOCT measured strut feature size with overlying thin and thick neointima (Fig. 6). Mean difference (4 μm) between strut size measured with IVOCT and Micro-CT for thin neointima is nearly zero and less than IVOCT lateral resolution. For thick neointima, mean difference (60 μm) between strut size measured with IVOCT and Micro-CT suggests a significant broadening of strut feature size in the presence of an overlying thick neointima.

Paired t-test for thin neointima measurements failed to reject the null hypothesis (*p* = 0.82) which suggests that IVOCT measurement of strut feature size in the presence of thin neointima on average match Micro-CT values. Paired t-test for thick neointima measurements rejected the null hypothesis (*p* < 0.001) which suggests that IVOCT measurement of strut feature size in the presence of thick neointima do not match Micro-CT values.

### Simulated neointima

**Fig. 7:**
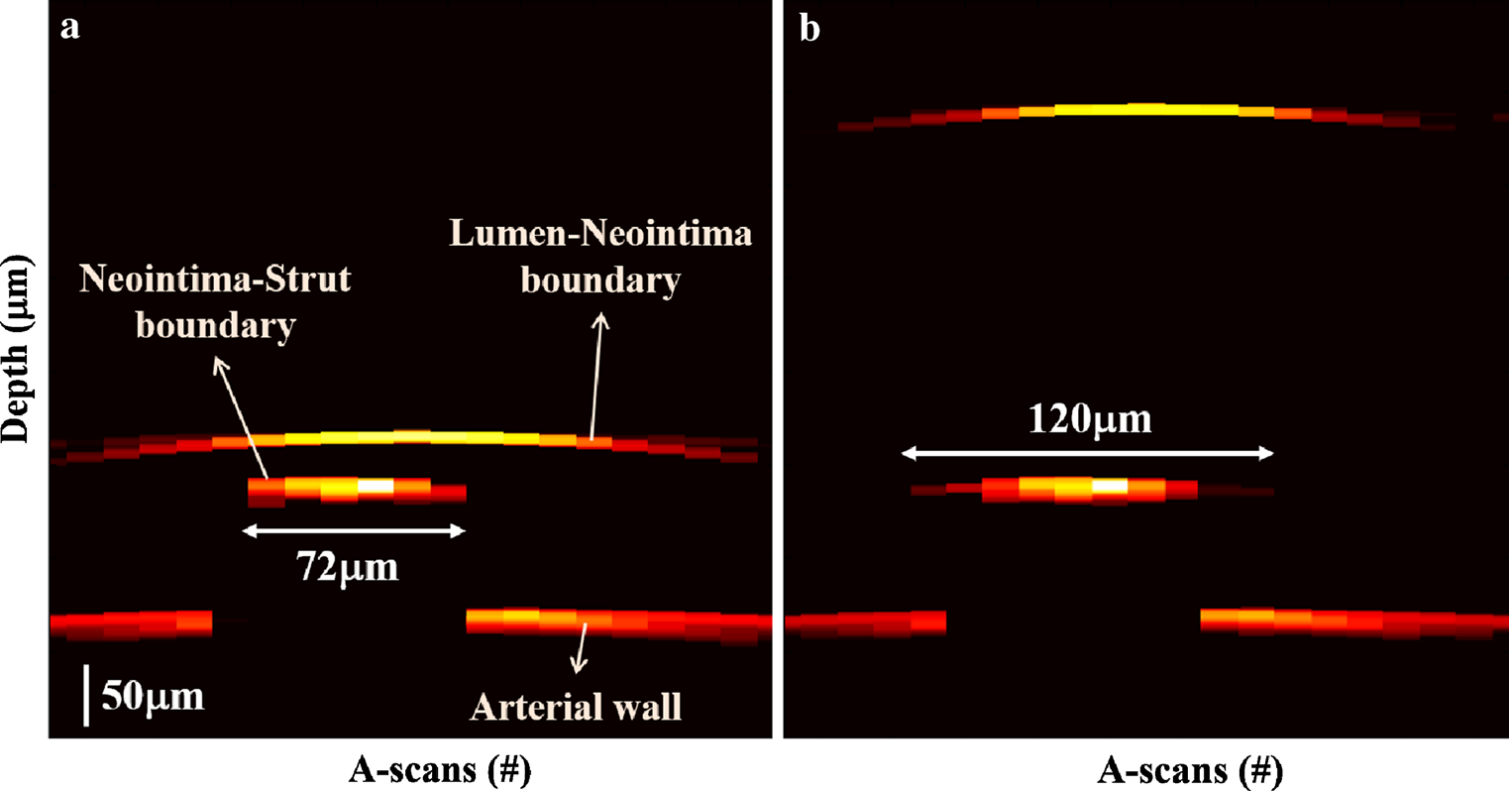
Simulated B-scans in presence of different neointimal thicknesses: **a** 50 μm, **b** 400 μm

Optical simulations were performed to investigate the effect of light scattering by neointima using user-defined more realistic optical properties; for example PDMS used to construct phantoms is isotropic (g ≈ 0) whereas tissue is forward scattering (g ≈ 0.8). Figure 7 illustrates effect of thin and thick neointima on appearance of the strut feature. Increase in neointimal thickness results in a merry-go-round artifact with an artifactual increase in the strut feature size of nearly 50 μm due to an overlying thick (400 μm) neointima. In this case incoming and reflected IVOCT light is scattered inside the neointima and then collected by neighboring A-scans adjacent to the actual strut thereby artifactually increasing strut feature size by 66.7 % in the IVOCT image.

## Discussion

IVOCT is the leading imaging modality for neointima detection and thickness measurements in vivo owing to the high resolution images it provides. The CorVue system utilized in this study is similar to existing commercial OCT systems (i.e., same center wavelength and similar imaging resolution) only operated at a slower A-scan rate (20 vs. 50 kHz). Reliability of IVOCT to assess the vascular response after metallic stent deployment where neointimal thickness is <80 microns has not been validated. Inasmuch as the presence of neointima after metallic stent deployment predicts lower risk of thrombosis, the ability to detect thin neointimas is an important factor to predict long-term patient outcome. In this study the accuracy of IVOCT neointimal thickness measurement was evaluated by comparing IVOCT images of vessels with phantom neointima covering metallic stents to actual values deduced from Micro-CT images. Bland–Altman plots of neointimal thickness indicate a relatively large variation in the difference between IVOCT and Micro-CT measurements. Variation in the difference between IVOCT and Micro-CT neointimal thicknesses can arise from errors in image registration and user placement of the line segment on corresponding images. Despite the measures taken to mitigate variations arising from errors in image registration, the disparate resolutions between IVOCT (25 μm lateral × 12 μm longitudinal) and Micro-CT (5.86 μm) together with a 50 μm spacing between successive IVOCT B-scans introduced a relatively large variation in the difference between IVOCT and Micro-CT measurements. Despite this relatively large variation, on average IVOCT measurement of thick neointimas are not statistically different from Micro-CT values (*p* > 0.05). For thin neointimas, mean difference (11 μm) between IVOCT and Micro-CT neointimal thickness is on the order of axial resolution (12 μm) of the CorVue system used to record IVOCT images and are statistically different from Micro-CT values (*p* < 0.05). The existence of a non-negative mean difference between IVOCT and Micro-CT values (thin: 11 μm, thick: 15 μm) suggests a systematic artifact may be present. A candidate mechanism for the observed non-negative mean difference might be the group refractive index value (1.38) utilized to convert optical pathlength to physical pathlength. Specifically, the value of group refractive index utilized may be too small and introduce the observed mean positive difference. In clinical practice, a number of uncertainties will complicate IVOCT measurement of neointimal thickness. Factors including the relative orientation between the IVOCT beam and neointimal surface normal and neointimal refractive index can contribute to increased variation between IVOCT and actual values. Because these factors can neither be controlled nor fully accounted for in clinical practice, IVOCT measurements of neointimal thickness should be evaluated with some caution. Results of this study suggest that relative variation of IVOCT measured neointimal thickness may vary from true values by as much as ±35 %.

The impact of luminal scattering on thickness measurement of thin neointimas was examined by using different concentrations of blood mixed with saline. Experimental data suggest that increased luminal scattering caused by residual blood concentrations <5 % in the lumen does not affect thickness measurement of thin neointimas. ANOVA statistical analysis confirms no significant variation (*p* = 0.95) exists between IVOCT neointimal thickness measurements at the four tested luminal blood scattering concentrations. The results suggest that in the clinic when residual blood (<5 %) remains in the lumen due to an incomplete flush, IVOCT measured neointimal thickness is not dependent on blood concentration.

The merry-go-round artifact in the presence of neointima was studied by phantom vessel experiments as well as optical simulations. In the case of thinner neointimas, metallic strut feature size measurements were not affected and IVOCT values match true values determined with Micro-CT. A paired t-test p-value (*p* = 0.82) supports the null hypothesis that on average IVOCT strut feature size measurements match Micro-CT values. Thicker neointimas, however, clearly introduce a relatively large (43 %) merry-go-round effect and a paired t-test p-value (*p* < 0.05) confirms IVOCT measured strut feature sizes in this case are significantly different than true values. Optical simulations support that neointimal scattering can introduce the merry-go-round effect; as neointimal layer becomes thicker, light reflecting from the strut surface undergoes multiple forward scattering events and are collected in adjacent neighboring A-scans. In this circumstance, metallic struts in the IVOCT image can appear elongated and the arterial wall may be observed behind the artifactually formed edges while shadowing is confined to the mid portion of the strut feature. Although the results presented here suggest size of the strut feature in IVOCT images may be difficult to interpret, ratio of size of the strut shadow to strut feature size in the absence of luminal scattering may provide a coarse measure of the composite scattering strength of the overlying neointima. Further studies will be required to investigate the potential diagnostic utility of analyzing this effect.
